# Clustering of *Vibrio parahaemolyticus* Isolates Using MLST and Whole-Genome Phylogenetics and Protein Motif Fingerprinting

**DOI:** 10.3389/fpubh.2019.00066

**Published:** 2019-05-08

**Authors:** Kelsey J. Jesser, Willy Valdivia-Granda, Jessica L. Jones, Rachel T. Noble

**Affiliations:** ^1^Institute of Marine Sciences, University of North Carolina at Chapel Hill, Morehead City, NC, United States; ^2^Orion Integrated Biosciences, New Rochelle, NY, United States; ^3^Gulf Coast Seafood Laboratory, Division of Seafood Science and Technology, U.S. Food and Drug Administration, Dauphin Island, AL, United States

**Keywords:** *Vibrio parahaemolyticus*, genomics, MLST, whole-genome sequencing, phylogenetics, protein motif fingerprinting, virulence

## Abstract

*Vibrio parahaemolyticus* is a ubiquitous and abundant member of native microbial assemblages in coastal waters and shellfish. Though *V. parahaemolyticus* is predominantly environmental, some strains have infected human hosts and caused outbreaks of seafood-related gastroenteritis. In order to understand differences among clinical and environmental *V. parahaemolyticus* strains, we used high quality DNA sequencing data to compare the genomes of *V. parahaemolyticus* isolates (*n* = 43) from a variety of geographic locations and clinical and environmental sample matrices. We used phylogenetic trees inferred from multilocus sequence typing (MLST) and whole-genome (WG) alignments, as well as a novel classification and genome clustering approach that relies on protein motif fingerprints (MFs), to assess relationships between *V. parahaemolyticus* strains and identify novel molecular targets associated with virulence. Differences in strain clustering at more than one position were observed between the MLST and WG phylogenetic trees. The WG phylogeny had higher support values and strain resolution since isolates of the same sequence type could be differentiated. The MF analysis revealed groups of protein motifs that were associated with the pathogenic MLST type ST36 and a large group of clinical strains isolated from human stool. A subset of the stool and ST36-associated protein motifs were selected for further analysis and the motif sequences were found in genes with a variety of functions, including transposases, secretion system components and effectors, and hypothetical proteins. DNA sequences associated with these protein motifs are candidate targets for future molecular assays in order to improve surveys of pathogenic *V. parahaemolyticus* in the environment and seafood.

## Introduction

*Vibrio parahaemolyticus* is a native member of bacterial flora in coastal ecosystems worldwide (1) and is a leading cause of illness associated with seafood (2, 3). *V. parahaemolyticus* bioaccumulates in oysters and other filter feeders during warm months and has been shown to proliferate rapidly in waters >15°C (4). When conditions are optimal for growth, virtually 100% of oysters have detectable concentrations of *V. parahaemolyticus* or other potentially pathogenic *Vibrio* species (5). Consumption of uncooked or mishandled seafood, often raw oysters, is a major mode of infection for *V. parahaemolyticus*, which causes an estimated 45,000 cases of gastrointestinal illness each year in the United States (6) and accounts for almost 50% of food poisoning outbreaks in Taiwan, Japan, and Southeast Asia (7, 8). Increasing rates of vibriosis have been reported around the world, especially at high latitudes where the increase has been correlated to rising sea surface temperatures (9–11). The prevalence of *V. parahaemolyticus* in densely populated coastal areas, as well as the economic value of the tourism and seafood industries, underscores the importance of accurate detection, quantification, and monitoring measures for this pathogen. However, measuring the abundance of disease-causing *V. parahaemolyticus* is difficult because most strains isolated from environmental sources are considered nearly exclusively environmental and nonpathogenic. Ecological and genomic similarities between known virulent strains and strictly environmental strains of *V. parahaemolyticus* make differentiating the organisms that actually cause disease challenging, but is vital when considering the risk *V. parahaemolyticus* populations may pose to human health (12).

Because not all strains of *V. parahaemolyticus* are considered truly pathogenic, putative virulence genes that are epidemiologically correlated with disease-causing strains are used to predict public health risks (13, 14). The *tdh* (thermostable direct hemolysin) and *trh* (thermostable-related hemolysin) genes are considered major virulence factors for *V. parahaemolyticus* (14), and many molecular detection methods, including several real-time quantitative PCR (qPCR) assays, have been developed based on these genes [e.g., (14–16)]. However, there have been multiple reports of clinical strains that do not have *tdh* and/or *trh* [e.g., (17–19)], contributing to concerns that these genes may not be reliable markers for virulence. The occurrence of *tdh* and *trh* in environmental isolates is typically 1–10% but can be much higher depending on sample location, source, and detection method, indicating that these genes may have environmental functions not related to human virulence (20). Other genetic markers that are thought to be important for pathogenicity include type-III secretion systems (T3SS) and effector proteins, urease genes, and genes involved in bacterial adherence and biofilm formation (21). Wagley et al. (22) showed that *tdh-*/*trh*- (nontoxigenic) strains were phylogenetically similar to other virulent *V. parahaemolyticus* strains despite not carrying virulence genes typically associated with disease-causing strains. This study also demonstrated that both nontoxigenic and toxigenic (*tdh*+ and/or *trh*+) strains can cause disease in the *Galleria mellonella* moth model, suggesting that there are unknown genes that are important for virulence in nontoxigenic strains.

Because of the challenges associated with detecting and quantifying pathogenic *V. parahaemolyticus* in the environment, understanding the relatedness of pathogenic and presumptive nonpathogenic environmental strains is key to gaining insights into the genomic differences between pathogenic and nonpathogenic *V. parahaemolyticus* strains. Molecular studies exploring the relatedness between *V. parahaemolyticus* isolates have employed a range of phylogenetic approaches, including single gene analyses (23, 24) and multilocus sequence typing (MLST) (25, 26) to investigate how clinical and environmental strains segregate into phylogenetic groups based on genetic similarity. MLST-based phylogenetic methods in particular have been used to illuminate the evolutionary patterns associated with the emergence of virulent phenotypes. However, despite being widely-used to study the molecular epidemiology of *V. parahaemolyticus*, MLST-based approaches, which rely on sequencing internal housekeeping gene loci, have limited phylogenetic resolution due to the relatively small amount of sequence data that is used. This is especially problematic for very similar strains or clonal populations which have caused disease outbreaks. For example, MLST phylogenetic methods are unable to differentiate ST36 strains, which have been tied to disease outbreaks associated with raw oyster consumption and improperly handled cooked shellfish in the United States and Europe (26–28).

Increasingly, phylogenetic studies are focused on genome-wide approaches, which have strain-level resolution and have become popular as whole-genome (WG) sequencing technologies have become less expensive and more widely available. A number of phylogenetic approaches which incorporate WG sequencing data have been utilized for *V. parahaemolyticus* and other bacterial pathogens. WG approaches for *V. parahaemolyticus* have relied on the alignment of core-genome genes, single nucleotide polymorphisms (SNPs), or draft or complete genome sequences (29–31). A study by Turner et al. (31) which relied on the alignment of WG sequences found enhanced phylogenetic resolution for pathogenic *V. parahaemolyticus* strains, which enabled the analysis of subclade diversity for pathogenic ST36 and ST3 strains. Whistler et al. (32) also used a WG method to achieve enhanced phylogenetic resolution for ST36 strains. Similar conclusions regarding the usefulness of WG-based phylogenetic analyses have been reached for a range of bacterial groups, including enterotoxigenic *E. coli* (33) and other bacterial species which are relevant to public health (30).

In the current study, we utilized high-quality WG shotgun sequencing data for 43 *V. parahaemolyticus* strains isolated from both clinical and environmental sample matrices between 2006 and 2010 from geographic locations in the United States and Prince Edward Island (PEI), Canada. We compared the relationships between strains using MLST and WG phylogenetic methods, as well as a novel classification approach based on protein motifs that are identified in raw sequencing data using DNA scanning algorithms (34). The protein motif method uses short protein fragments specific to a given taxonomic group or functional category to reveal relationships between bacterial pathogens and molecular markers associated with virulence. Together, a group of protein motifs constitutes a motif fingerprint (MF) for an isolate that can be used to identify genomic regions associated with pathogenicity. The MF method is based on the knowledge that each bacterial species and strain has distinctive short protein-coding sequences that can be used to distinguish between and classify microorganisms (35), and has recently been used alongside phylogenetics to investigate the molecular evolution of epizootic hemorrhagic disease viruses (36, 37). Importantly, MFs are not limited to functional or virulence genes, and MF clustering is not limited to vertically transferred phylogenetic relationships. Instead, each MF is specific to a pathogen family, genus, species, or strain, and may incorporate multiple individual protein motifs, thus avoiding biases or assumptions commonly associated with the identification of gene targets associated with bacterial pathogens. Additionally, this MF approach is advantageous since it can be used to screen, scan, and directly compare raw sequencing datasets without the need for a genome assembly step. The objective of this research was to compare the results of MLST and WG phylogenetic clustering with MF clustering for both clinical and environmental *V. parahaemolyticus* isolates. We demonstrate that the phylogenetic and MF clustering methods are complementary and describe how MF clustering can be used to identify molecular targets associated with virulent *V. parahaemolyticus* strains.

## Materials and Methods

### Strain Information and Typing

A total of 43 *V. parahaemolyticus* strains were used in this study. Metadata, including strain serovar, MLST type, location and year isolated, sample matrix, and *tdh*/*trh* typing, for all strains is listed in Table 1. Raw WG shotgun sequencing data were downloaded from the NCBI SRR database for 4 *V. parahaemolyticus* strains (NCBI BioSamples SAMN01923894, SAMN01940374, SAMN02741394, and SAMN02741402). Raw WG shotgun sequencing data and associated metadata for an additional 39 strains were provided by the Food and Drug Administration (FDA) Gulf Coast Seafood Laboratory (GCSL). Data for the FDA strains, which were sequenced as part of the University of California at Davis 100K Pathogen Genome Project, are also available in NCBI (see NCBI BioSample IDs in Table 1). *V. parahaemolyticus tdh*/*trh* gene presence or absence was determined for the FDA strains at the FDA GCSL using the protocol described in Nordstrom et al. (14) for multiplex qPCR with an internal amplification control. Serotyping of the FDA isolates was as described in (17). For strains where raw sequence data were downloaded directly from NCBI, *tdh*/*trh* gene presence or absence data was collected from previously published studies (39, 40), with the exception of SAMN01923894. To our knowledge, SAMN01923894 has not been PCR-typed for *tdh* or *trh*, though we did find a *tdh* homolog in a RASTtk (41) annotation of the draft genome. However, because the genome is not closed, we cannot be sure whether *trh* is truly absent. For this reason, SAMN01923894 is listed in Table 1 and in all figures as “not typed.”

**Table 1:** *Vibrio parahaemolyticus* isolates.

	**NCBI BioSample ID^a^**	**Matrix**	**Year^b^**	**Location^b^**	**Serovar**	**Sequence type (ST)**	***tdh/trh***
1	SAMN02368229	Oyster	2007	FL	O4:Kuk	536	–/–
2	SAMN02368232	Oyster	2007	FL	O11:Kuk	734	–/–
3	SAMN02368266	Oyster	2007	FL	O4:K42	1146	–/–
4	SAMN02368267	Oyster	2007	FL	O11:Kuk	1153	–/–
5	SAMN02368274	Oyster	2007	FL	O5:Kuk	743	–/–
6	SAMN02368227	Oyster	2007	LA	O4:K10	732	–/–
7	SAMN03358821	Oyster	2007	PEI, Canada	O11:Kuk	1152	–/–
8	SAMN02368264	Oyster	2007	PEI, Canada	O11:Kuk	1152	–/–
9	SAMN02368270	Oyster	2007	SC	O3:Kuk	741	–/–
10	SAMN02368244	Oyster	2007	WA	O3:Kuk	1148	–/–
11	SAMN02741394	Oyster	2010	MD	Unk	34	–/+
12	SAMN02741402	Oyster	2010	MD	Unk	8	–/+
13	SAMN02368293	Stool	2006	HI	O4:K4	283	–/–
14	SAMN02368297	Stool	2006	MA	O4:K53	749	+/+
15	SAMN02368298	Stool	2006	MA	O1:Kuk	3	+/–
16	SAMN02368290	Stool	2006	MD	O5:K47	1144	–/+
17	SAMN02368282	Stool	2006	ME	O5:Kuk	1150	–/+
18	SAMN03358827	Stool	2006	NY	O10:Kuk	636	+/+
19	SAMN03358828	Stool	2006	NY	O3:K6	3	+/–
20	SAMN02368284	Stool	2006	NY	O4:Kuk	36	+/+
21	SAMN02368283	Stool	2006	NY	O10:Kuk	809	–/+
22	SAMN03358830	Stool	2006	NY	O4:K12	36	+/+
23	SAMN02368288	Stool	2006	VA	O8:K41	Undefined^c^	–/–
24	SAMN02368286	Stool	2006	VA	O5:K17	674	–/–
25	SAMN02368315	Stool	2007	AK	O4:K63	36	+/+
26	SAMN02368321	Stool	2007	GA	O4:K8	Undefined^c^	+/–
27	SAMN02368292	Stool	2007	HI	O5:Kuk	79	–/–
28	SAMN02368291	Stool	2007	HI	O5:K17	79	–/–
29	SAMN02368322	Stool	2007	IA	O4:K12	36	+/+
30	SAMN02368323	Stool	2007	IA	O4:K12	36	+/+
31	SAMN02368304	Stool	2007	MD	O3:K56	750	+/+
32	SAMN03358834	Stool	2007	NV	O1:Kuk	199	+/+
33	SAMN03358837	Stool	2007	NY	O10:Kuk	636	+/+
34	SAMN03358839	Stool	2007	OR	O1:Kuk	65	–/+
35	SAMN02368303	Stool	2007	SD	O1:K56	775	+/+
36	SAMN02368318	Stool	2007	VA	O1:K20	1132	+/+
37	SAMN02368312	Stool	2007	WA	O4:K12	36	+/+
38	SAMN02368311	Stool	2007	WA	O4:K12	36	+/+
39	SAMN02368325	Stool	2007	WA	O4:Kuk	36	+/+
40	SAMN02368333	Stool	2009	OK	O4:K12	36	+/+
41	SAMN01923894	Unk	2006	USA	Unk	3	Not typed
42	SAMN01940374	Water	2009	USA	Unk	1567	–/–
43	SAMN02368278	Hand	2006	LA	O1:Kuk	744	–/–

a *BioSample IDs are searchable in NCBI's BioSample database; web entries include sample information and links to raw sequence data*.

b *Indicates year/location of collection for environmental isolates and year/location of sample isolation from patient for clinical isolates*.

c *ST is undefined due to an insertion in the recA MLST locus [strains with similar insertions described in (38)]*.

### Genome Assembly

Raw sequencing reads were trimmed prior to genome assembly using the JGI bbduk tool (k = 27, ktrim = l, hdist = 1, minlength = 50). High-quality draft genomes were assembled using SPAdes v. 3.10.0 (42) or Velvet v. 1.2.10 (43) prokaryotic genome assemblers as implemented in Geneious v. 11.0.4 (44). K-mer sizes for the SPAdes assemblies were selected automatically by the software and the careful option was selected to reduce mismatches and short indels. Velvet assemblies were run using the manual option with k-mer size = 33. The best assembly was evaluated based on N50 values and the number and length of assembled contigs. Based on these criteria, either the Velvet or SPAdes assembly was selected for each isolate. Contigs < 200 bp in length were filtered from the assemblies. Genome scaffolding was done using the Medusa web server (45) with all closed *V. parahaemolyticus* genomes in NCBI (*n* = 19; accessed January 2018) used as comparison genomes. Genome assembly statistics are listed in Table S1.

### MLST Phylogenetic Tree Building

MLST loci were extracted *in silico* from assembled genomes using the online tool at the Center for Genomic Epidemiology [CGE, https://cge.cbs.dtu.dk/services/MLST/; (46)], which obtains MLST allele sequence and profile data from PubMLST.org (47). The MLST scheme used for *V. parahaemolyticus* was first published by Gonzalez-Escalona et al. (48), and relies on internal sequences of 7 housekeeping gene loci which span both of the *V. parahaemolyticus* chromosomes (*recA, dnaE, gyrB, dtdS, pntA, pyrC*, and *tnaA*). MLST sequences were downloaded from CGE's webservice and imported into Geneious. In Geneious, MLST sequences were concatenated and a 3,682 bp sequence alignment was built using MUSCLE v. 3.8.425 (49). A maximum-likelihood phylogenetic tree was inferred using RAxML v. 8.2.11 (50) as implemented in Geneious with the general time-reversible gamma substitution model. RAxML tree-building started with a complete random tree and the best scoring maximum likelihood tree was selected after 1,000 replicates of rapid bootstrapping. Rapid bootstrapping was also used to calculate branch support values for the MLST tree. Two strains, SAMN02368288 and SAMN02368321, had undefined STs due to insertions in the *recA* MLST locus [see (38) for a description of similar strains]. These strains were not included in the MLST alignment or tree.

### WG Phylogenetic Tree Building

Scaffolds for *V. parahaemolyticus* isolates were aligned using Mugsy, an aligner for closely-related genomes which identifies collinear regions using a segment-based progressive multiple alignment system (51). The Mugsy WG alignment was converted from maf to fasta format with one entry per genome using the Galaxy web-platform's converter tool, which joins and converts conserved alignment blocks shared by all genomes in the alignment (52). TrimAL (53) with the strictplus algorithm was used to remove spurious and poorly aligned positions and divergent regions in the WG alignment, with a resulting core sequence alignment of 4,629,130 bp. An approximate maximum-likelihood phylogenetic tree was inferred using FastTree v. 2.1.5 (54) with the general time-reversible model. The reliability of each split in the phylogenetic tree was assessed using both FastTree support values, which are based on the Shomodaira-Hasegawa test of three alternate topologies around each split, and 1,000 bootstrap replicates, which were generated using PHYLIP SEQBOOT software (55). Both MLST and WG phylogenetic trees were midpoint rooted and annotated using the ggtree package (56) in R v. 3.5.1 (57).

### Protein Motif Fingerprint Discovery

Motif discovery analyses were performed on Orion Integrated Biosciences servers using MF generation (MF-gen) and CHAST algorithms to identify protein fragments associated with precise taxonomies via an exhaustive search of GenBank protein databases as described in Corpas et al. (58) and Wilson et al. (36). Briefly, all protein entries in GenBank were divided into 12-amino acid subsequences (motifs) that were position-independent and did not contain overlaps. Every known proteome across >6.7 million taxonomies assigning organism strain, serotype, species, family, and superfamily were searched against this motif library and each motif was assigned a detailed and specific taxonomic label. In addition, we used a library of MFs covering >600,000 plasmids. We classified three types of MFs: (i) MF-type I are segments specific to a given taxonomic group (e.g., *Vibrio* species or *V. parahaemolyticus* strains), (ii) MF-type II are shared by the host and pathogen only, and may have been co-opted by the pathogen to influence immune signaling or regulatory/metabolic pathways, and (iii) MF-type III are non-specific segments shared in more than two species. Only MF-types I and II were used to scan the raw, unassembled sequence reads of the 43 *V. parahaemolyticus* isolates via perfect matching after a 6-frame translation process.

### MF Clustering

*Vibrio*-associated motifs were selected and manually filtered for those that were highly variable across the 43 *V. parahaemolyticus* genomes. Motif abundance counts were normalized across isolates into a matrix using Genesis v. 1.7.7 (59), where the presence of an MF in each bacterial genome was presented in an MF event matrix (MFEM) where each row (*g*) represented a normalized MF occurrence count and each column represented an MF event (*n*) in a given strain. The distance between strains was determined in Genesis as described in (36), where the MFEM = *g* × *n* array was clustered using an average linkage hierarchical clustering algorithm using the Pearson correlation coefficient (*r*) between *g* and *n*. Select protein motif sequences associated with genome clusters of interest were assigned putative gene functions using blastp (60).

## Results

A total of 43 high-quality draft genomes of *V. parahaemolyticus* strains isolated in the United States and PEI, Canada between 2006 and 2010 were compared using both MLST and WG phylogenetics and a novel protein motif clustering analysis. Of these 43 genomes, 29 (67.4%) were isolated from clinical matrices (human samples), 13 (30.2%) were isolated from water or oysters, and 1 was from an unknown sample matrix. All but one isolate was assayed for the presence of *tdh* and *trh* genes using PCR. Of the 42 isolates for which *tdh*/*trh* typing was done, 17 (40.4%) were *tdh*+/*trh*+, 3 (7.1%) were *tdh*+/*trh*–, 5 (11.9%) were *tdh*–/*trh*+, and 17 (40.4%) were *tdh*–/*trh*–. Of the 29 clinical isolates, 16 (55.1%) were *tdh*+*/trh*+, 3 (10.3%) were *tdh*+*/trh*–, 4 (13.8%) were *tdh*–*/trh*+, and 6 (20.7 %) were *tdh*–*/trh*–. Serotyping of the 39 FDA isolates revealed 20 unique serotypes. Isolate metadata is summarized in Table 1.

### MLST

*In silico* MLST analyses revealed 30 unique MLST types, indicating a high degree of genetic diversity amongst strains. There were 2 strains that had *recA* insertions that resulted in undefined MLST types. The number of MLST types covered in this study is comparable to others which have focused on isolates from North America, though the number of MLST types reported varies depending on the number of isolates analyzed and the geographic and temporal range of the study [see (26, 61, 62) for relevant examples]. The most common ST types associated with the 29 clinical isolates included in the present study were ST36 (31.0%), and ST3 (10.3%), both of which have been associated with outbreaks of gastrointestinal illness (63, 64) and have been reported as common clinical MLST types (26). MLST-*tdh/trh* typing results in the present study are aligned with previous findings. For example, ST36 isolates included in this study were found to be *tdh*+*/trh*+, as has been previously described for ST36 strains (28, 61). MLST types for all isolates are summarized in Table 1.

### Phylogenetic Trees

**Figure 1:**
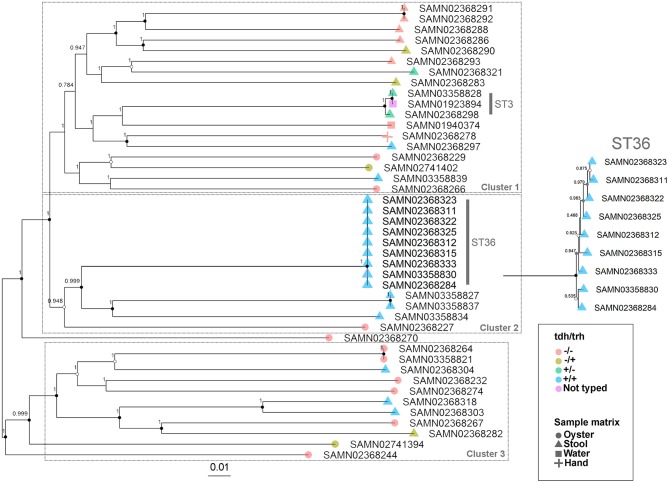
Approximate maximum-likelihood phylogenetic tree based on a whole genome (WG) sequence alignment of 43 *V. parahaemolyticus* genomes. Colors indicate hemolysin gene (*tdh* and *trh*) presence or absence and shape indicates sample isolation source. Inset is an enlargement of the ST36 clade to illustrate subclade diversity and resolution. Nodes are labeled with FastTree support values. Circles at nodes indicate bootstrap support values (1000 replicates) of >0.9 (black), >0.7 (gray), and >0.5 (white). Numbered clusters correspond with clusters listed in Table 2.

**Figure 2:**
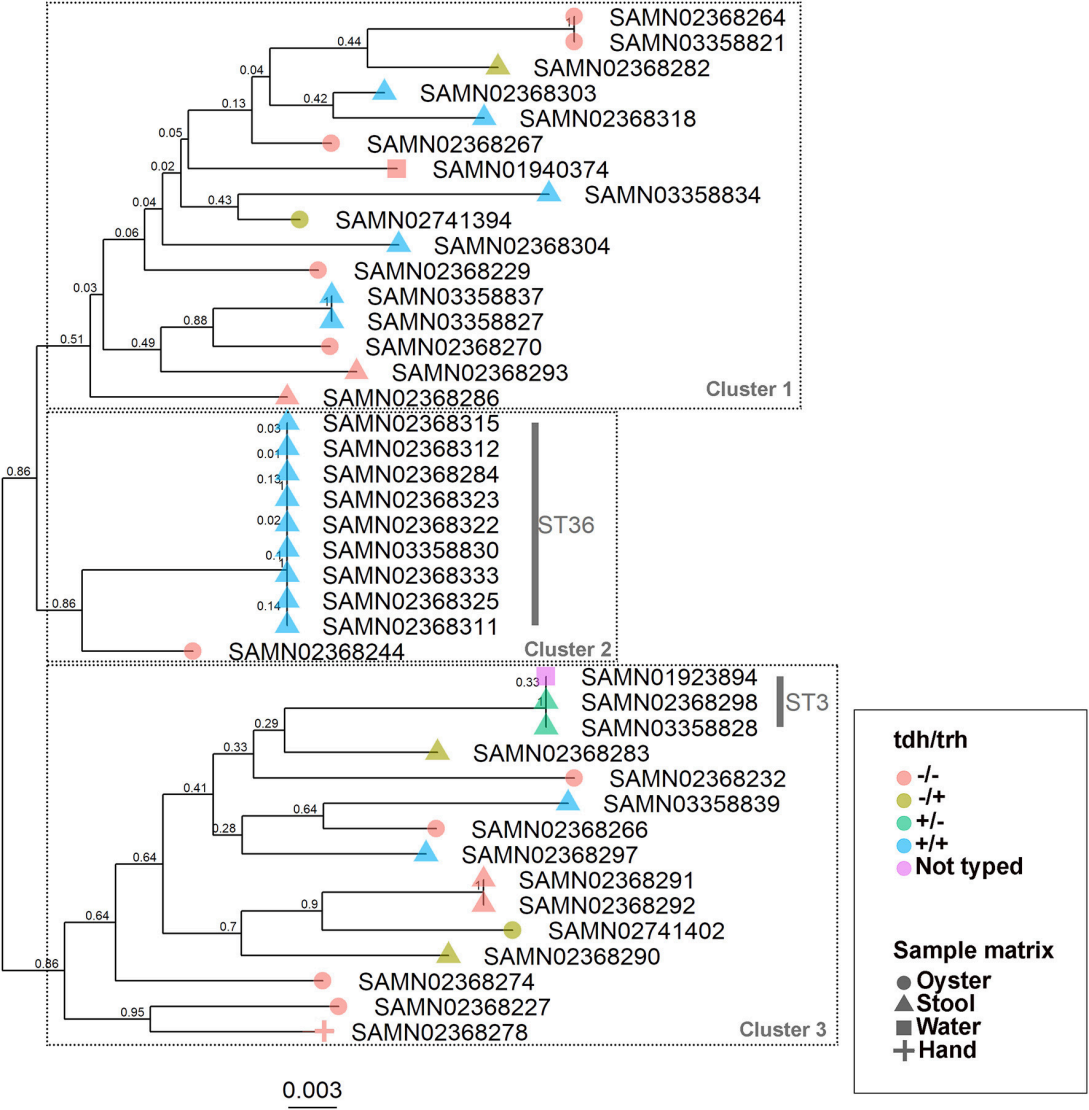
Maximum-likelihood phylogenetic tree based on an alignment of multilocus sequence typing (MLST) loci for 43 *V. parahaemolyticus* genomes. Colors indicate hemolysin gene (*tdh* and *trh*) presence or absence and shape indicates sample isolation source. Nodes are labeled with bootstrap support values (1000 replicates). Numbered clusters correspond with clusters listed in Table 2.

The WG phylogenetic tree (Figure 1), which was inferred using a 4,629,130 bp multiple sequence alignment, had improved taxonomic resolution in comparison to the MLST phylogenetic tree (Figure 2), which was inferred using a 3,682 bp multiple sequence alignment. The largest clusters in the phylogenetic trees were labeled (Figures 1, 2) and membership of the *V. parahaemolyticus* strains in these clusters is summarized in Table 2. The WG tree was fully bifurcating, but the MLST tree contained relationships that were not fully resolved because MLST sequences for isolates with the same MLST type were identical. The WG phylogeny had improved branch support values at most nodes. Based on visual inspection, no clear trends tying *tdh*/*trh* typing to clustering results were observed in the MLST phylogenetic tree. In the WG phylogenetic tree, clinical nontoxigenic strains were clustered together and with *tdh*–/*trh*+ strains. The *tdh*+*/trh*+ clinical strains also largely clustered together in the WG phylogeny, though some *tdh*+/*trh*+ clinical strains grouped more closely with environmental, nontoxigenic strains. WG trees annotated with serovar, MLST type, and the location/year strains were isolated are available in the supplementary material (Figures S1–S3).

**Table 2:** Cluster membership of *Vibrio parahaemolyticus* isolates in the WG and MLST phylogenies and the MF clustering analysis.

	**NCBI BioSample ID**	**WG cluster^a^**	**MLST cluster^b^**	**MF cluster^c^**
1	SAMN02368229	1	1	
2	SAMN02368232	3	3	
3	SAMN02368266	1	3	
4	SAMN02368267	3	1	
5	SAMN02368274	3	1	
6	SAMN02368227	2	3	
7	SAMN03358821	3	1	
8	SAMN02368264	3	1	
9	SAMN02368270		1	
10	SAMN02368244	3	2	
11	SAMN02741394	3	1	
12	SAMN02741402	1	3	
13	SAMN02368293	1	1	
14	SAMN02368297	1	3	Stool cluster
15	SAMN02368298	1	3	
16	SAMN02368290	1	3	Stool cluster
17	SAMN02368282	3	1	Stool cluster
18	SAMN03358827	2	1	Stool cluster
19	SAMN03358828	1	3	
20	SAMN02368284	2	2	ST36
21	SAMN02368283	1	3	Stool cluster
22	SAMN03358830	2	2	ST36
23	SAMN02368288	1	N/A^d^	Stool cluster
24	SAMN02368286	1	1	
25	SAMN02368315	2	2	ST36
26	SAMN02368321	1	N/A^d^	
27	SAMN02368292	1	3	Stool cluster
28	SAMN02368291	1	3	Stool cluster
29	SAMN02368322	2	2	ST36
30	SAMN02368323	2	2	ST36
31	SAMN02368304	3	1	
32	SAMN03358834	2	1	Stool cluster
33	SAMN03358837	2	1	Stool cluster
34	SAMN03358839	1	3	Stool cluster
35	SAMN02368303	3	1	Stool cluster
36	SAMN02368318	3	1	Stool cluster
37	SAMN02368312	2	2	ST36
38	SAMN02368311	2	2	ST36
39	SAMN02368325	2	2	ST36
40	SAMN02368333	2	2	ST36
41	SAMN01923894	1	3	
42	SAMN01940374	1	1	
43	SAMN02368278	1	3	

a *Corresponds to numbered clusters in the WG phylogeny (Figure 1)*.

b *Corresponds to numbered clusters in the MLST phylogeny (Figure 2)*.

c *Corresponds to labeled MF clusters (Figure 3)*.

d *Isolate not included in MLST analysis due to an insertion in the* recA *MLST locus*.

### MF Clustering

Hierarchical clustering was used to group genomes and protein motifs (Figure 3). A large group of stool isolates was identified in the protein motif analysis (Figure 3, cluster indicated in green) that included 22 of 29 (75.9%) clinical strains. Of these stool cluster isolates, 15 (68.2%) were *tdh*+, 19 (86.4%) were *trh*+, and 3 (13.6%) were nontoxigenic. The stool genome cluster was defined by two protein motifs. One of these, assigned the specific taxonomic label “O29774: *V. parahaemolyticus* (TH3996),” was present in all but one of the stool-associated genomes in the group. Motifs with this taxonomic label were found in the coding sequences of several hypothetical proteins, transposases, a hemolysin, and putative proteins associated with the cell membrane, conjugation, and the T3SS apparatus and effectors (see Table S2 for the list of top blast hits).

**Figure 3:**
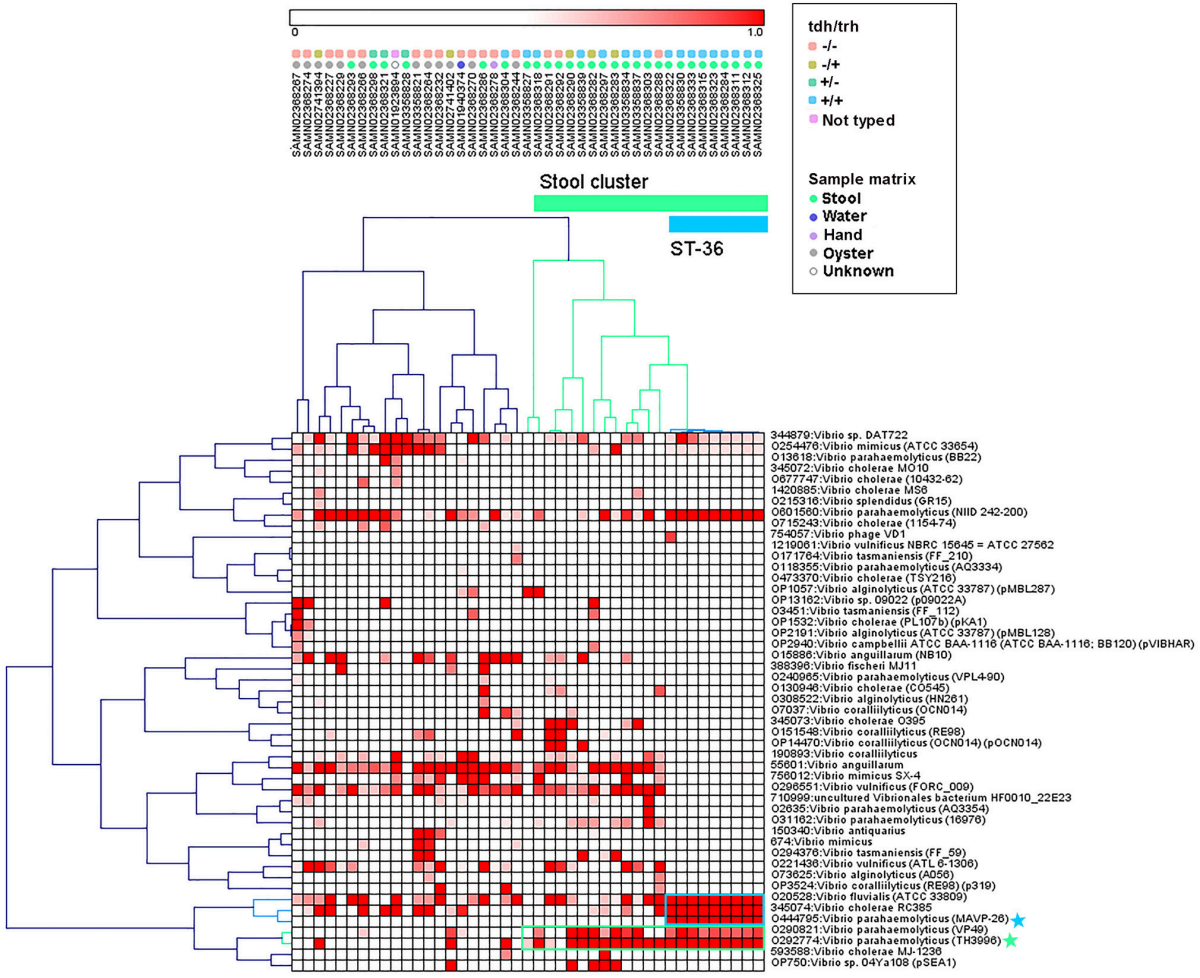
Pearson correlation average linkage hierarchical clustering of *Vibrio* motif fingerprints (MFs) across 43 *V. parahaemolyticus* genomes revealed a large cluster of stool isolates (green) and clustered ST36 isolates together (blue). Protein motifs associated with these clusters are designated by colored boxes, and specific motif taxonomies that are proposed as targets for quantitative molecular assay design are labeled with colored stars.

Within the MF stool cluster, we observed a group of 9 strains with very similar MFs that corresponded to ST36 (Figure 3, cluster indicated in blue), a clonal sequence type that is *tdh*+/*trh*+ and has caused widespread human illness (65). This ST36 genome cluster was defined by a small group of protein motifs, one of which, labeled “O444795: *V. parahaemolyticus* (MAVP _26),” was specific to the ST36 genomes. Motifs associated with this taxonomic label were identified in the coding sequences of a wide array of functional genes, including hypothetical proteins, transcriptional regulators, transporter proteins, an RTX toxin, and a capsular biosynthesis protein (Table S2). Membership of individual *V. parahaemolyticus* isolates in either the stool-associated or the ST36 MF clusters is summarized in Table 2.

## Discussion

### Comparison of WG and MLST Phylogenetic Trees

We observed differences in strain clustering at more than one position between the phylogenetic trees inferred using the WG and MLST sequence alignments (Figures 1, 2). This has been previously observed for *V. parahaemolyticus* (31) and multiple other bacterial species (30). Phylogeny inferred using MLST loci, while reproducible, discriminatory, and potentially useful for epidemiological surveillance and disease outbreak investigations, cannot fully represent genome phylogeny because it utilizes relatively little sequence data and because MLST gene loci do not exemplify the entire genome. For these reasons, MLST trees should be interpreted with caution. In the present study, the amount of sequence data represented by the MLST alignment (3,682 bp) was just 0.08% of that utilized in the WG (4,629,130 bp) alignment. The lower branch support values computed for the MLST tree compared to the WG tree are likely due to the relatively small number of informative sites in the MLST alignment. However, because MLST-based analyses have been so widely used in epidemiological studies of *V. parahaemolyticus*, MLST typing information is still essential to interpret WG sequence data in the context of previous research and disease outbreaks.

The importance of the improved taxonomic resolution of the sequence-based WG phylogeny is exemplified by the fully bifurcating trees for strains of the same sequence type (see Table 1 for MLST sequence types and Figure 1 for the WG phylogeny). Though little differentiation was observed for the 9 ST36 strains in the WG tree, the WG phylogenetic approach did allow us to observe relationships between strains that we were unable to discern in the MLST phylogeny. These strain-level differences for very closely-related strains are key to understanding the evolution of pathogenic types as they move through the environment and human populations. A previous WG study of *V. parahaemolyticus* (28) found that ST36 strains from a disease outbreak in Maryland could be differentiated from historical strains isolated on the west coast of the United States using a genome-wide SNP phylogenetic analysis. These results, as well as the results of the current study, underscore the value of fully-resolved WG phylogenetic trees for *V. parahaemolyticus* as a means to define the diversity and evolution of disease-causing types.

The present study corroborates previous findings on the usefulness of WG phylogenetic approaches for *V. parahaemolyticus* (31, 32). However, although WG sequencing methods are becoming increasingly affordable and accessible, it is important to note that the computational capacity required to utilize WG sequence alignments to infer phylogenetic relationships of closely related bacterial strains may be prohibitive. For example, constructing the alignment used to infer the WG phylogenetic tree for the 43 *V. parahaemolyticus* isolates presented in the current study took approximately 10 hours and 9 GB memory on a high-performance computing cluster. In contrast, the MLST alignment took <1 min and was computed locally on a desktop computer. Tree building and bootstrapping also required significantly more time and memory for the WG vs. the MLST phylogenetic analysis. As computational capacity and knowledge are both increasing in the field, WG alignments will likely become more feasible in the future. In the meantime, other phylogenetic methods which rely on WG sequencing data can be used. For example, phylogenies inferred using SNPs have been suggested as an alternative to WG sequence alignments because they cover the entire genome and are less time and resource-intensive. Though we did not construct a SNP-based phylogenetic tree in the present study, previous research has shown similar tree topologies and branch support values for SNP and WG phylogenies for several bacterial pathogens (30). A core genome MLST (cgMLST) method has also been reported for *V. parahaemolyticus* (29). We did not construct a phylogeny using cgMLST loci in the present study, but this method has been shown to produce fast typing results and meaningful, high resolution phylogenies using coding sequences identified in WG datasets.

### MF Clustering

The protein MF clustering results provided fresh insights into the genomic characteristics of *V. parahaemolyticus* strains as they relate to virulence gene presence/absence and isolation source which were unbiased by previous assumptions about strain pathogenicity. Hierarchical clustering of MFs associated with select *Vibrio* taxa (Figure 3) produced two genome clusters of particular interest. The first was a large cluster of clinical *V. parahaemolyticus* strains isolated from human stool (Figure 3, cluster indicated in green). The second was a cluster of genomes that corresponded to ST36 (Figure 3, cluster indicated in blue), that had very similar MF profiles to one another and were observed within the larger stool-associated cluster.

The large stool-associated cluster included ~75% of clinical *V. parahaemolyticus* isolates in our dataset. Though this large cluster of clinical isolates was mostly composed of toxigenic strains, the *tdh*/*trh* profiles did not align perfectly with the MF clustering or clinical isolation sources. Approximately 25% of the clinical isolates were not associated with the stool-related MF cluster, and the clinical strains that did not group with the stool-related cluster had MF profiles more similar to environmental strains. Most of the clinical isolates that were not in the stool-related cluster were nontoxigenic, but 4 were *tdh*+ and/or *trh*+. Conversely, three of the isolates that were in the stool-associated cluster were nontoxigenic. Together, these results support the idea that *tdh* and *trh* gene presence may not be sufficient for defining pathogenic strains because there are unknown genomic factors associated with virulence. On the other hand, broadly speaking, most isolates in the stool-associated cluster did have either *tdh* (~70%) and/or *trh* (~85%) and, based on visual inspection, the MF analysis more clearly clustered strains based on *tdh*/*trh* virulence gene typing than was observed in either the MLST or WG phylogenetic trees. Despite some caveats, the presence of *tdh*/*trh* hemolysin genes did seem to be associated with genome-wide differences linked to virulence in the MF analysis.

The second MF cluster of interest was a group of *tdh*+/*trh*+ ST36 genomes, which were clustered together within the larger stool-associated cluster. Given the genomic similarity and phylogenetic relatedness of ST36 strains (Figure 1) it was unsurprising that these strains also clustered in the MF analysis. We consider this an excellent example of the complementarity of the MF analysis with the phylogenetic and MLST methods we used, because we may not have recognized this cluster as ST36 without the MLST results or immediately realized how closely related these strains were if we had not done the phylogenetic analyses. The fact that the ST36 MF cluster was found within the larger stool-associated cluster was interesting, since this pattern was not observed in the WG phylogeny (Figure 1). Based on the MF patterns we observed, it seems that ST36 strains may have genomic signatures of virulence which are also present in other, non-ST36 pathogenic strains.

### Future Directions: MFs to Quantitative Molecular Assays

In order to utilize the MF data for the protection of shellfish consumers, the next step in this line of research is to use the sequences of protein motifs associated with the specific genome clusters we identified in the MF analysis to define genomic indicators of virulent *V. parahaemolyticus* strains. The genome regions containing protein motifs, and indeed the motif sequences themselves, can then be targeted for the development of novel qPCR assays. The hemolysin genes *tdh* and *trh* are currently the most commonly used markers for virulent *V. parahaemolyticus* in seafood and the environment. As previously discussed, these markers are useful for predicting the abundance of virulent *V. parahaemolyticus*, but the emergence of nontoxigenic clinical strains that do not carry either hemolysin gene is cause for concern. Using the MF approach, we believe we have the capacity to develop qPCR primers and probes that can be used to improve predictions of *V. parahaemolyticus* virulence potential by capturing some of these nontoxigenic but pathogenic strains. Assays designed using protein motif targets could be used in conjunction with current qPCR methodologies to improve predictions of virulence potential. Though protein motif fingerprinting of bacterial isolates is a novel and powerful bioinformatics tool, the process of developing quantitative assays from protein motif sequences is nontrivial because each protein motif taxonomic label is associated with hundreds to thousands of unique motif sequences. In order to design a functional gene assay using motif sequences, motifs must first be searched against public databases for gene functions and cross-reactivity. The next step is to ensure that the DNA coding regions which include the protein motif sequences are suitable for PCR assay designs by filtering them based on their thermodynamic characteristics. Once suitable motifs have been selected, they can be used to design qPCR primers and probes.

## Conclusions

As next-generation sequencing technologies continue to advance, it is possible that sequence-based methods could one day become fully quantitative and rapid enough to completely replace PCR-based quantitative methods for environmental and-seafood related surveys of virulent *V. parahaemolyticus*. In the meantime, our results suggest that MF clustering shows great promise for identifying new genomic indicators of virulent strains that are not constrained by previous ideas about *V. parahaemolyticus* virulence traits. Future assays developed using specific motif sequences could be used to improve predictions of potentially pathogenic *V. parahaemolyticus* in the environment and shellfish and contribute to improved public health outcomes.

## Author Contributions

The original idea for this project was developed by KJ, RN, and WV-G. KJ analyzed the data and drafted the manuscript with input and final approval from WV-G, JJ, and RN. WV-G conducted the initial protein motif fingerprinting motif analyses and provided input on the evaluation of the resultant data. JJ and the FDA GCSL contributed genomic sequencing data and associated metadata used in the analyses.

### Conflict of Interest Statement

The motif fingerprinting software was developed by WV-G from Orion Integrated Biosciences. The remaining authors declare that the research was conducted in the absence of any commercial or financial relationships that could be construed as a potential conflict of interest.
