# Supplementary material for: Clustering of Vibrio parahaemolyticus Isolates Using MLST and Whole-Genome Phylogenetics and Protein Motif Fingerprinting
Source: Front Public Health. 2019 May 8;7:66. doi: 10.3389/fpubh.2019.00066 (PMC6519141; doi:10.3389/fpubh.2019.00066)
Supplement: Supplementary file 1 [file Data_Sheet_1.docx]

**Supplementary Figures and Tables**

| **Table S1. Vp genome assembly statistics** | | | | | | | | | | | | |
| --- | --- | --- | --- | --- | --- | --- | --- | --- | --- | --- | --- | --- |
| **NCBI BioSample ID** | **Read length (bp)** | **Number of reads^a^** | **Assembler** | **Contigs >200 bp** | **Contig N50**  **length (bp)** | **Contigs >N50** | **Mean contig length (bp)** | **Longest contig (bp)** | **Number of scaffolds** | **Scaffold N50 length (bp)** | **Sequencing depth^b^** | **% Genome completeness^c^** |
| SAMN02741394 | 45-220 | 7.26E5 | SPAdes | 375 | 2.98E4 | 51 | 1.31E4 | 1.20E5 | 28 | 2.68E6 | 22x | 97 |
| SAMN02741402 | 50-300 | 1.11E6 | SPAdes | 354 | 3.22E4 | 50 | 1.37E4 | 1.26E5 | 40 | 1.59E6 | 42x | 99 |
| SAMN01923894 | 100 | 3.66E6 | SPAdes | 182 | 2.60E5 | 6 | 1.87E4 | 7.58E5 | 26 | 1.43E6 | 144x | 99 |
| SAMN01940374 | 100 | 1.25E7 | SPAdes | 66 | 1.93E5 | 6 | 8.40E3 | 7.19E5 | 9 | 2.55E6 | 501x | 97 |
| SAMN02368315 | 100 | 1.22E7 | SPAdes | 678 | 3.32E5 | 5 | 7.42E3 | 8.58E5 | 283 | 8.51E5 | 232x | 101 |
| SAMN02368229 | 100 | 5.32E6 | SPAdes | 124 | 3.60E4 | 5 | 3.01E4 | 6.53E5 | 26 | 1.36E6 | 106x | 96 |
| SAMN02368232 | 100 | 3.49E6 | SPAdes | 103 | 3.73E5 | 5 | 3.10E4 | 8.43E5 | 24 | 1.70E6 | 72x | 93 |
| SAMN02368266 | 100 | 9.66E6 | SPAdes | 115 | 2.18E5 | 7 | 2.92E4 | 6.38E5 | 23 | 1.44E6 | 188x | 99 |
| SAMN02368267 | 100 | 6.20E6 | SPAdes | 111 | 2.27E5 | 8 | 2.80E4 | 4.02E5 | 19 | 2.92E6 | 127x | 94 |
| SAMN02368274 | 100 | 2.36E7 | SPAdes | 104 | 3.28E5 | 5 | 2.75E4 | 8.64E5 | 28 | 1.74E6 | 481x | 94 |
| SAMN02368321 | 100 | 8.25E6 | SPAdes | 207 | 5.39E5 | 4 | 2.01E4 | 8.71E5 | 33 | 1.42E6 | 162x | 98 |
| SAMN02368293 | 100 | 4.99E6 | Velvet | 74 | 2.59E5 | 6 | 6.68E4 | 5.22E5 | 10 | 3.19E6 | 101x | 95 |
| SAMN02368291 | 100 | 3.27E6 | SPAdes | 105 | 3.17E5 | 6 | 3.01E4 | 5.80E5 | 20 | 3.86E6 | 65x | 97 |
| SAMN02368292 | 100 | 3.62E6 | SPAdes | 121 | 3.02E5 | 6 | 2.66E4 | 5.80E5 | 29 | 1.77E6 | 72x | 97 |
| SAMN02368322 | 100 | 4.55E6 | SPAdes | 119 | 5.48E5 | 4 | 3.32E4 | 8.58E5 | 42 | 3.23E6 | 88x | 99 |
| SAMN02368323 | 100 | 6.18E6 | SPAdes | 131 | 5.48E5 | 4 | 3.02E4 | 8.58E5 | 39 | 1.80E6 | 121x | 99 |
| SAMN02368227 | 100 | 1.25E7 | SPAdes | 142 | 3.16E5 | 6 | 2.49E4 | 6.19E5 | 42 | 9.56E5 | 246x | 98 |
| SAMN02368278 | 100 | 6.54E6 | SPAdes | 128 | 2.24E5 | 7 | 2.82E4 | 8.20E5 | 28 | 1.58E6 | 127x | 99 |
| SAMN02368297 | 100 | 8.69E6 | SPAdes | 167 | 3.16E5 | 6 | 2.22E4 | 5.64E5 | 38 | 1.21E6 | 169x | 99 |
| SAMN02368298 | 100 | 5.51E6 | SPAdes | 136 | 2.59E5 | 7 | 2.50E4 | 6.53E5 | 18 | 2.77E6 | 108x | 98 |
| SAMN02368290 | 100 | 4.22E6 | SPAdes | 102 | 3.50E5 | 5 | 3.23E4 | 3.50E5 | 22 | 1.82E6 | 81x | 100 |
| SAMN02368304 | 100 | 7.04E6 | SPAdes | 103 | 2.67E5 | 7 | 3.09E4 | 5.31E5 | 25 | 1.39E6 | 139x | 97 |
| SAMN03358834 | 100 | 1.91E6 | SPAdes | 126 | 3.13E5 | 6 | 2.58E4 | 5.47E5 | 24 | 2.99E6 | 38x | 98 |
| SAMN03358827 | 100 | 3.48E6 | SPAdes | 142 | 2.09E5 | 7 | 2.95E4 | 5.59E5 | 36 | 1.76E6 | 65x | 102 |
| **Table S1 cont. Vp genome assembly statistics** | | | | | | | | | | | | |
| **NCBI BioSample ID** | **Read length (bp)** | **Number of reads^a^** | **Assembler** | **Contigs >200 bp** | **Contig N50**  **length (bp)** | **Contigs >N50** | **Mean contig length (bp)** | **Longest contig (bp)** | **Number of scaffolds** | **Scaffold N50 length (bp)** | **Sequencing depth^b^** | **% Genome completeness^c^** |
| SAMN03358828 | 100 | 5.80E6 | SPAdes | 217 | 2.68E5 | 6 | 1.93E4 | 6.53E5 | 103 | 1.24E6 | 113x | 98 |
| SAMN02368282 | 100 | 4.90E6 | SPAdes | 121 | 2.07E5 | 6 | 2.70E4 | 8.65E5 | 23 | 1.78E6 | 99x | 95 |
| SAMN02368283 | 100 | 4.13E6 | SPAdes | 84 | 2.08E5 | 6 | 2.97E4 | 6.37E5 | 14 | 3.05E6 | 81x | 98 |
| SAMN03358830 | 100 | 5.93E6 | SPAdes | 124 | 5.48E5 | 4 | 3.27E4 | 5.48E5 | 34 | 1.07E6 | 117x | 98 |
| SAMN02368284 | 100 | 5.22E6 | SPAdes | 116 | 5.48E5 | 4 | 3.09E4 | 8.58E5 | 27 | 1.07E6 | 103x | 98 |
| SAMN03358837 | 100 | 2.30E6 | SPAdes | 122 | 2.10E5 | 6 | 3.13E4 | 5.59E5 | 18 | 1.35E6 | 45x | 98 |
| SAMN02368333 | 100 | 8.82E6 | SPAdes | 117 | 5.48E5 | 4 | 3.27E4 | 7.95E5 | 27 | 1.24E6 | 173x | 98 |
| SAMN03358839 | 100 | 2.35E6 | SPAdes | 97 | 3.13E5 | 6 | 4.34E4 | 5.71E5 | 15 | 1.07E6 | 46x | 99 |
| SAMN03358821 | 100 | 3.87E6 | SPAdes | 115 | 2.47E5 | 8 | 3.31E4 | 4.91E5 | 29 | 1.34E6 | 79x | 95 |
| SAMN02368264 | 100 | 9.16E6 | SPAdes | 118 | 2.35E5 | 8 | 3.25E4 | 4.91E5 | 24 | 1.34E6 | 186x | 95 |
| SAMN02368270 | 100 | 3.35E6 | SPAdes | 176 | 5.37E5 | 4 | 2.23E4 | 7.55E5 | 64 | 1.73E6 | 66x | 97 |
| SAMN02368303 | 100 | 6.50E6 | SPAdes | 152 | 1.49E5 | 11 | 2.24E4 | 4.37E5 | 29 | 2.88E6 | 130x | 96 |
| SAMN02368286 | 100 | 7.19E6 | SPAdes | 103 | 4.73E5 | 4 | 2.79E4 | 8.80E5 | 15 | 1.76E6 | 141x | 98 |
| SAMN02368288 | 100 | 3.87E6 | Velvet | 112 | 3.15E5 | 6 | 4.63E4 | 5.37E5 | 33 | 1.78E6 | 75x | 99 |
| SAMN02368318 | 100 | 5.09E6 | SPAdes | 151 | 1.30E5 | 11 | 1.69E4 | 4.05E5 | 15 | 1.73E6 | 101x | 97 |
| SAMN02368244 | 100 | 6.68E6 | SPAdes | 117 | 2.64E5 | 7 | 2.75E4 | 5.23E5 | 19 | 1.82E6 | 131x | 98 |
| SAMN02368311 | 100 | 7.99E6 | SPAdes | 479 | 4.03E5 | 5 | 1.01E4 | 8.67E5 | 172 | 8.68E5 | 155x | 99 |
| SAMN02368312 | 100 | 9.30E6 | SPAdes | 113 | 4.06E5 | 5 | 3.46E4 | 3.67E5 | 20 | 1.61E6 | 183x | 98 |
| SAMN02368325 | 100 | 3.97E6 | SPAdes | 116 | 3.22E5 | 5 | 3.01E4 | 8.58E5 | 29 | 3.17E6 | 79x | 97 |
| ^a^after bbduk trimming  ^b^Estimated using the Lander/Waterman method where coverage=(avg. read length x read number)/genome size  ^c^Where % completeness=total length of scaffolded contigs/5.2e6 genome size for Vp ref strain RIMD2210633 *100 | | | | | | | | | | | | |

| **Table S2. Top blast hits of motif sequences associated with MF clusters of interest** | |
| --- | --- |
| Protein motif taxonomic label | Top blast hits for protein motifs^1^ |
| O29774: *Vibrio* parahaemolyticus (TH3996)^2^ | hemolysin D |
|  | hypothetical proteins (11) |
|  | putative integral membrane protein |
|  | putative T3SS effector protein vop3 (cytotoxin) |
|  | putative TraA (conjugal transfer protein) |
|  | putative T3SS apparatus protein |
|  | transposases (4) |
| O444795: *Vibrio* parahaemolyticus (MAVP_26)^3^ | acetate-coA ligase |
|  | alcohol dehydrogenase |
|  | alpha-1,2-fucosyltransferase |
|  | capsular biosynthesis protein |
|  | cell division protein/translocase FtsK |
|  | chromosome partitioning protein  (cobQ/cobB/MinD/ParA nucleotide binding  domain protein) |
|  | DksA/TraR family C4-type zinc finger protein |
|  | DUF3404 domain-containing protein |
|  | flippase |
|  | fructose-6-phosphate aldolase |
|  | GGDEF domain-containing protein |
|  | glycosyltransferase family 1 protein |
|  | GNAT family N-acetyltransferase |
|  | HNH endonuclease |
|  | hypothetical proteins (7) |
|  | MFS transporter |
|  | molybdate ABC transporter substrate-binding protein |
|  | peptidase S8 |
|  | putative mannitol repressor domain protein |
|  | RTX toxin |
|  | S9 family peptidase |
|  | tRNA His guanylyltransferase family protein (2) |
|  | XRE family transcriptional regulator |
| ^1^Number of hits indicated in parentheses  ^2^Taxonomic label for protein motif which defined the large stool-related cluster of Vp isolates  ^3^Taxonomic label for protein motif which defined ST36 cluster of Vp isolates | |

**Figure S1.** Phylogenetic tree based on a whole genome (WG) sequence alignment. Colored text indicates isolate serovar. Nodes are labelled with FastTree support values.


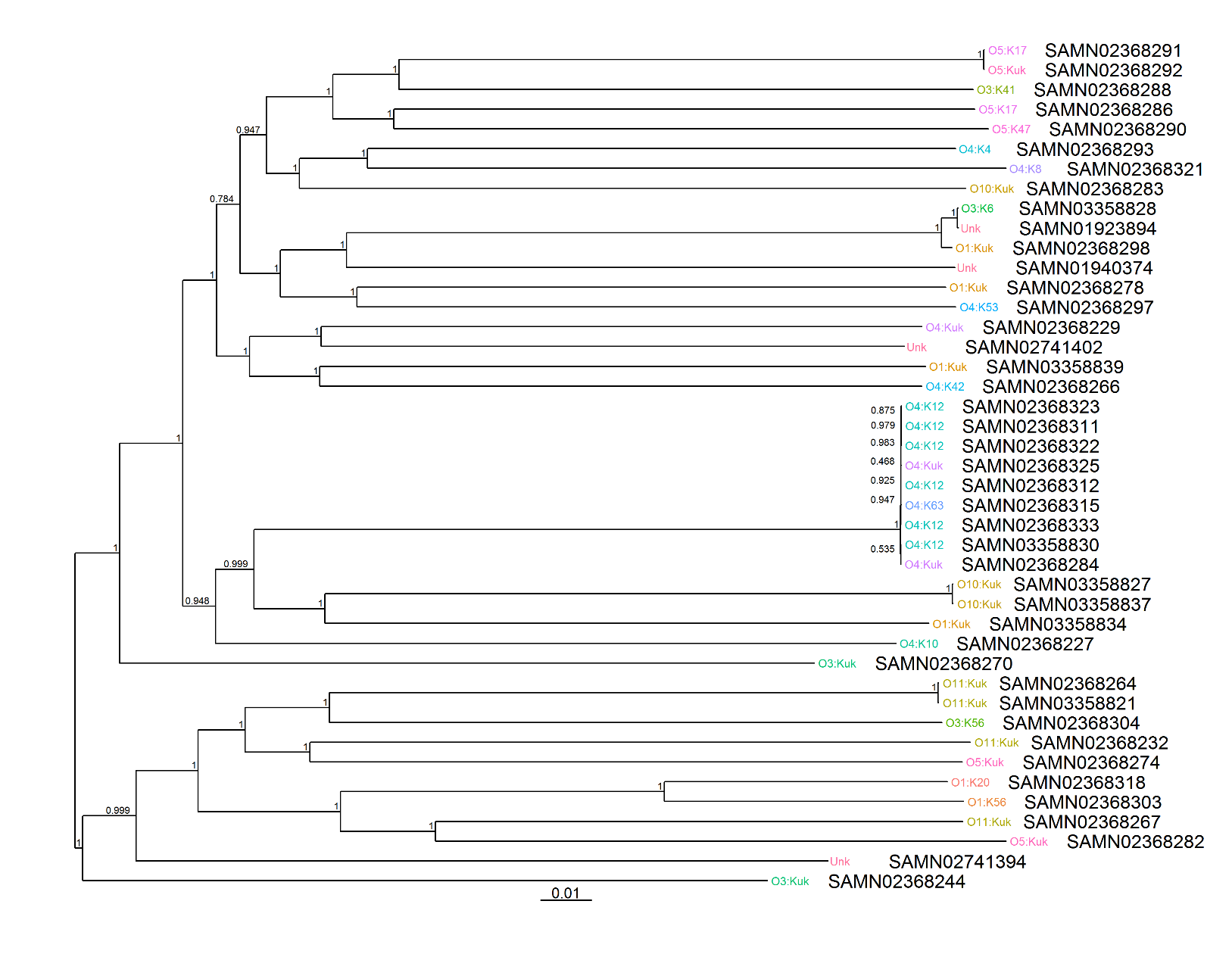

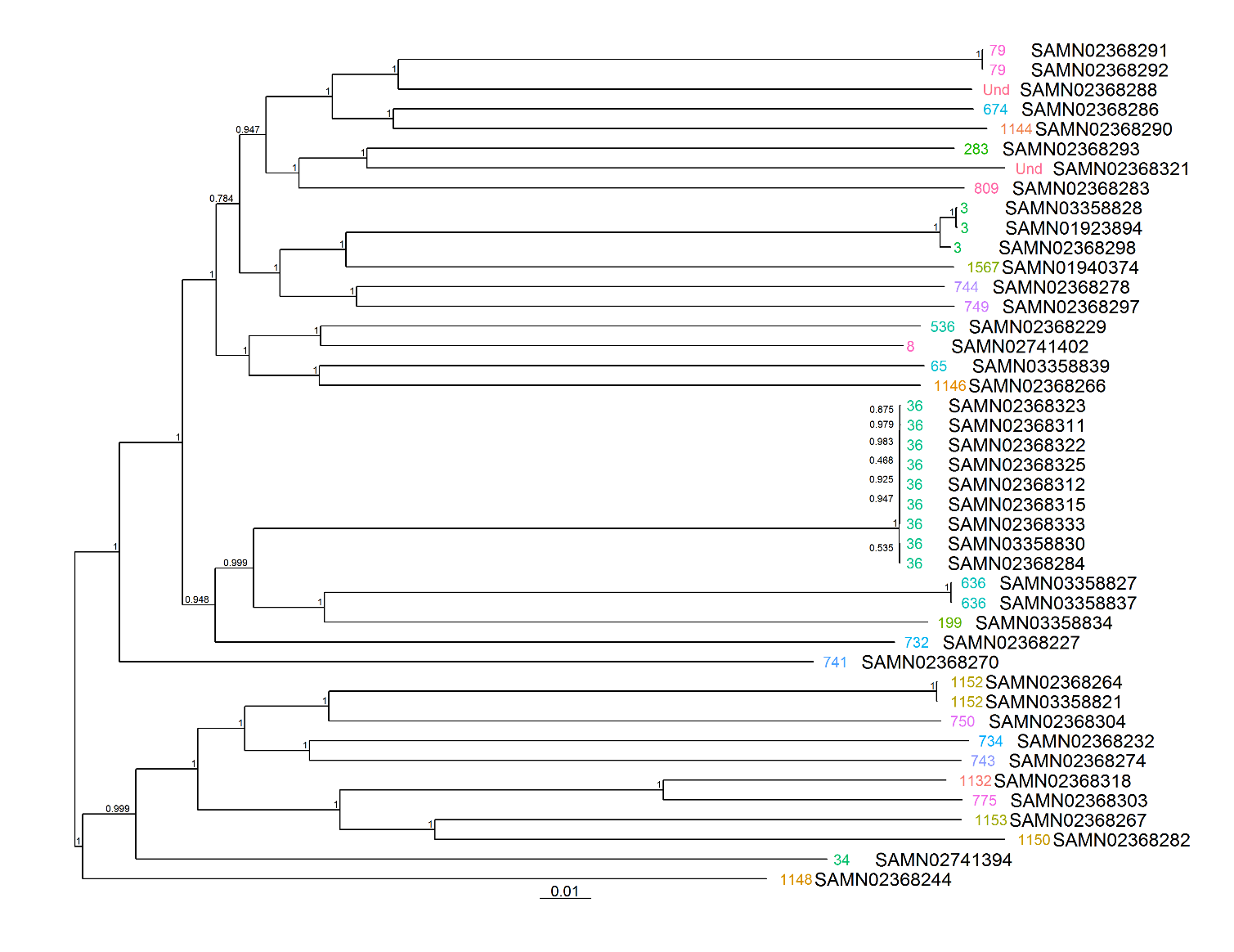


**Figure S2.** Phylogenetic tree based on a whole genome (WG) sequence alignment. Colored text indicates isolate MLST type. Nodes are labelled with FastTree support values.


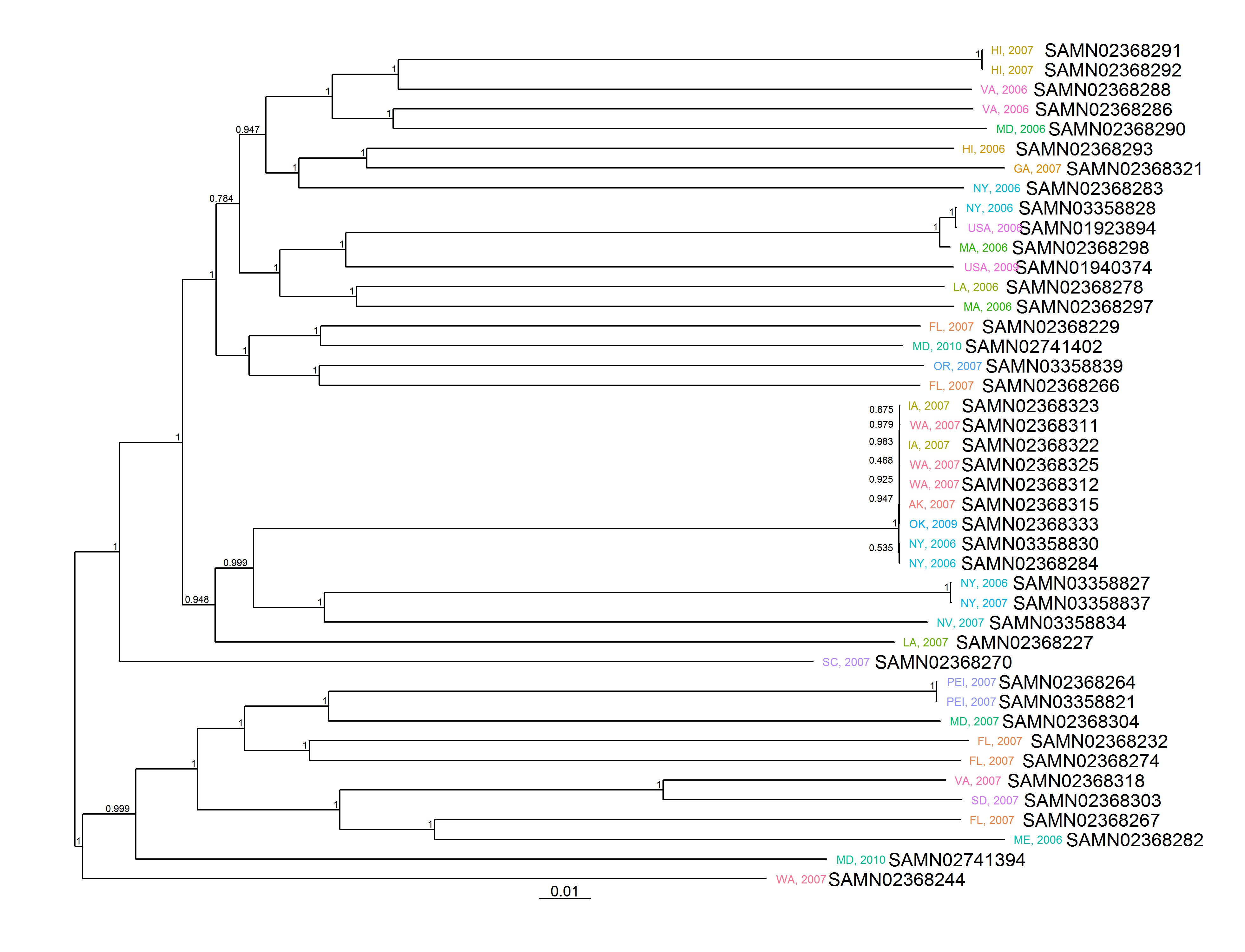


**Figure S3.** Phylogenetic tree based on a whole genome (WG) sequence alignment. Colored text indicates location (state or Canadian province) and year strain was isolated. Nodes are labelled with FastTree support values.
